# The Interplay of Hypertension and Anemia on Pregnancy Outcomes

**DOI:** 10.7759/cureus.46390

**Published:** 2023-10-02

**Authors:** Alby Johnson, Sasi Vaithilingan, Seetha Lakshmi Avudaiappan

**Affiliations:** 1 Department of Obstetrics and Gynaecological Nursing, Vinayaka Mission’s Research Foundation (DU), Salem, IND; 2 Department of Child Health Nursing, Vinayaka Mission's College of Nursing, Puducherry, IND; 3 Department of Nursing Foundation, Sri Ramachandra College of Nursing, Sri Ramachandra Institute of Higher Education and Research, Chennai, IND

**Keywords:** newborn outcomes, maternal outcomes, pregnancy complications, anemia, pregnancy-induced hypertension

## Abstract

Background

Anemia and pregnancy-induced hypertension (PIH) are two significant high-risk conditions that can have a profound impact on maternal health during pregnancy. The scarcity of studies investigating the potential links and interactions between these two conditions adds to the gap in our understanding of their combined impact on pregnant women. This study aimed to assess the impact of PIH in conjunction with anemia and without anemia on pregnancy outcomes.

Methods

A prospective study was conducted among 150 primi pregnant women (third trimester of pregnancy) from a secondary-care hospital (Government General Headquarters Hospital, Cuddalore) in Tamil Nadu, India. The study population was selected using a purposive sampling technique. Data were collected using a structured questionnaire to assess sociodemographic characteristics, dietary patterns, and outcomes of pregnancy. Clinical parameters such as blood pressure were measured using a sphygmomanometer, and hemoglobin and urine tests for protein were obtained from the patient’s medical records.

Results

The result showed that out of 150 primi pregnant women, 73 (49%) had PIH and 77 (51%) experienced both PIH and anemia. On comparing the outcomes, pregnant women with PIH accompanied by anemia exhibited developing preeclampsia (p<0.001), encountered maternal complications (p=0.034), delivered preterm babies (p=0.03), and gave birth to low-birth-weight babies (p<0.001), and their newborns admitted to the NICU (p=0.02). Additionally, pregnant women with both PIH and anemia demonstrated significantly higher levels of systolic blood pressure after delivery (p=0.009).

Conclusion

The study calls for the immediate attention of healthcare providers for vigilant monitoring and addressing anemia in conjunction with PIH to improve maternal and neonatal outcomes.

## Introduction

Maternal and newborn health during and after pregnancy is the most urgent reproductive health issue in the world [1]. A recent report by the United Nations shows that approximately 4.5 million maternal and newborn deaths occur - one death every 7 seconds among which most of them are preventable [2]. India has contributed 12% to maternal deaths with the leading cause being obstetric hemorrhage with underlying causes such as anemia and pregnancy-induced hypertension (PIH) [3,4]. Anemia during pregnancy is a pervasive concern on a global scale [5] and poses a substantial public health challenge in India, affecting a significant proportion of expectant mothers who have hemoglobin levels below 11 g/dL. The high prevalence of anemia has grave implications, as it serves as either the primary cause or a contributing factor in 20-40% of maternal deaths that occur within the country [6]. Anemia refers to a decrease in the number of red blood cells or a decrease in the amount of hemoglobin (Hb). Pregnancy-related anemia is often caused by iron deficiency, but it can also result from other factors such as vitamin deficiencies or chronic diseases. During pregnancy, it can lead to perinatal complications, including preeclampsia, postpartum hemorrhage, premature birth, and low birth weight [7,8]. PIH, on the other hand, refers to high blood pressure, which can arise during pregnancy and is categorized as gestational hypertension and preeclampsia [9]. Gestational hypertension is characterized by high blood pressure (BP ≥ 140/90 mmHg) that develops after 20 weeks of gestation without the presence of other complications, whereas preeclampsia is a more severe form of hypertension that is typically accompanied by the presence of protein in the urine indicating organ damage [10]. A study reported that 5-15% of pregnancies in India are affected by PIH [11]. Hypertensive disorders of pregnancy can increase the risk of preterm birth, fetal growth restriction, placental abruption, and other adverse outcomes for both mother and newborn [12].

When PIH coincides with anemia, the interaction between these two conditions introduces unique challenges and doubles the risks to both the mother and the fetus [13]. Anemic women with hypertension may experience a more severe form of preeclampsia and are at a higher risk of developing complications such as placental abruption and maternal organ damage. Additionally, the combination of anemia and hypertension leads to poor fetal growth and development, increasing the likelihood of preterm birth and low birth weight [14]. Despite its significance to maternal health, there remains a scarcity of studies that have comprehensively synthesized evidence on the relationship between these two high-risk conditions. This lack of research further highlights the importance of exploring and understanding how anemia and PIH may interact and potentially exacerbate each other's effects during pregnancy and its outcome. Recognizing its importance, the present study was carried out to determine the impact of PIH in conjunction with anemia on pregnancy outcomes.

## Materials and methods

This was a prospective study of primi pregnant women diagnosed with PIH accompanied by anemia or only PIH. The study was carried out in the Government Headquarters Hospital, Cuddalore, which is a secondary-care hospital providing Comprehensive Emergency Obstetric and Newborn Care (CEmONC). The Institutional Ethical Committee of Vinaya Mission's College of Nursing approved our study (VMCN PDY/IEC 2022/065) and it was conducted from August 2022 to January 2023. All primi pregnant women diagnosed with PIH accompanied by anemia or only PIH who satisfied the inclusion criteria were recruited using a purposive sampling technique. The study included primi pregnant women who were diagnosed with PIH, with or without anemia, and were receiving antenatal care services at the Obstetric and Gynecological Department of the Government Headquarters Hospital during their third trimester of pregnancy. Pregnant women diagnosed with any other medical illness apart from PIH and those who were unwilling to participate in the study were excluded. Participants included in the study were informed about the study and written consent was obtained from them. Participants who were between 29 weeks and 33 weeks of gestation were interviewed using a structured questionnaire. The participants of the study were followed in the immediate postpartum period.

The researcher developed a structured questionnaire to collect the data. The questionnaire consists of three parts. The first part consisted of socio-demographic questions that included details regarding age, education, working status, socioeconomic class, religion, type of family, history of hypertension in the family, and habits of consuming aerated drinks, tea, coffee, and milk (nine items). The second part had details on clinical variables such as body mass index (BMI), systolic blood pressure (SBP), diastolic blood pressure, and hemoglobin and urine tests for protein (five items). The third part dealt with the maternal and newborn outcomes including the development of preeclampsia, mode of delivery, blood pressure, maternal complications, birth weight of the baby, gestational week of baby, APGAR scores, admissions to NICU, and maternal and neonatal mortality (11 items). The constructed questionnaire was validated by the subject experts, and its reliability was checked using Cronbach’s alpha test (r=0.87). 

The height and weight of each primi pregnant woman were measured, and the metric system was used to calculate their Body Mass Index. The BMI of 18.5 to 24.9 was considered as normal, < 18.5 - underweight, 25 to 29.9 - overweight, and >30 - obesity (WHO Classification). The standard procedure was followed for measuring the blood pressure of the pregnant women. The systolic and diastolic blood pressure was measured manually using a sphygmomanometer. The pregnant women were asked to rest for five minutes and measured their BP three times at 2-minute interval. All measurements were recorded manually and averaged them. BP ≥ 140/90 mmHg was considered as hypertension and classified based on FOGSI-GESTOSIS-ICOG guidelines [15]. The data on the level of hemoglobin, urine test for protein, and maternal complications such as abruptio placenta, preeclampsia, eclampsia, pulmonary edema, and postpartum hemorrhage were collected from the medical records of the participants. Levels of hemoglobin were classified as normal - ≥11 g/dL, mild anemia - 10 - 10.9 g/dL, moderate anemia-7 - 9.9 g/dL, and severe anemia-<7 g/dL). The urine test for protein was considered negative if the presence of protein is <10 mg/dL, trace -10-20mg/dL, 1+ - 30 mg/dL, 2+ - 100 mg/dL, 3+ - 300 mg/dL, and 4+ - >1000 mg/dL. The EpiInfo version 7.0 was used to enter the data and IBM SPSS Statistics for Windows, Version 28 (Released 2021; IBM Corp., Armonk, New York, United States) was used to analyze the collected data and depicted in the form of tables and graphs. The categorical data were presented in frequencies and percentages. The continuous data were given as mean and standard deviation. In the analysis of outcome, an independent sample t-test was used to compare the continuous variables, whereas the Chi-square test was used to compare the categorical variables. The level of significance was set at a P value of <0.05. Odds ratios (ORs) and their corresponding 95% confidence intervals (CIs) were calculated.

## Results

A total of 150 primi pregnant women were included in the study, where 73 (49%) had PIH and 77 (51%) had both PIH and anemia. The mean age of pregnant women in the PIH without anemia group was found to be 25.30 ± 4.34 years and 24.58± 4.26 years in the PIH with anemia group. The minimum age was 18 years and the maximum age was 37 years in PIH without anemia group and in the PIH with anemia group the minimum age was 18 years and the maximum age was 38 years. In both groups, the majority of the participants had intermediate or post-high school diplomas (47% & 57%). More than half of the participants were not working (51% & 56%) and fit into the upper-lower socioeconomic class (56% & 57%). The majority of the participants in both groups belonged to the Hindu religion (84%) and were living in a nuclear family (88% & 87%). Most of the participants in both groups had no history of hypertension in the family. The habit of consuming aerated drinks was more common in both groups and the majority of them were taking tea/coffee/milk once a day (Table 1).

**Table 1:** Demographic characteristics of study participants PIH: Pregnancy-induced hypertension

Variables		*PIH without Anemia (n=73) f (%)	*PIH with Anemia (n=77) f (%)	p-value
Age	<20	4 (6%)	8 (10%)	0.51
	20-24	32 (44%)	39 (51%)	
	25-29	25 (34%)	18 (23%)	
	30- 34	9 (12%)	10 (13%)	
	≥35	3 (4%)	2 (3%)	
Education	School Certificate	9 (12%)	9 (12%)	0.39
	Intermediate or post-high school diploma	34 (47%)	44 (57%)	
	Graduate or above	30 (41%)	24 (31%)	
Working status	Non-working	37 (51%)	43 (56%)	0.53
	Working	36 (49%)	34 (44%)	
Socioeconomic class	Upper middle	15 (21%)	13 (17%)	0.82
	Lower middle	17 (23%)	20 (26%)	
	Upper lower	41 (56%)	44 (57%)	
Religion	Hindu	61 (84%)	65 (84%)	0.70
	Muslim	6 (8%)	8 (10%)	
	Christian	6 (8%)	4 (5%)	
Type of Family	Nuclear Family	64 (88%)	67 (87%)	0.90
	Joint Family	9 (12%)	10 (13%)	
History of hypertension in the family	No	58 (80%)	56 (73%)	0.33
	Yes	15 (21%)	21 (27%)	
Habit of consuming aerated drinks	No	27 (37%)	32 (42%)	0.57
	Yes	46 (63%)	45 (58%)	
Consumption of tea/coffee/milk	Once	39 (53%)	42 (55%)	0.89
	Twice or more	34 (47%)	35 (45%)	

In comparison, the mean BMI of the pregnant women was similar in both groups, 25.38 ± 3.34 versus 25.52 ±2.81, and was observed to be in the overweight range. In addition, the mean hemoglobin level was significantly lower in the PIH with anemic pregnant women (10.31 g/dL) which indicates the presence of anemia (p < 0.001) (Table 2). 

**Table 2:** Clinical parameters of study participants PIH: Pregnancy-induced hypertension

Clinical Variables	PIH without Anemia Mean ± SD (n=73)	PIH with Anemia Mean ± SD (n=77)	Independent sample t-test	p-value
Body Mass Index	25.38 ± 3.34	25.52 ±2.81	0.27	0.79
Systolic Blood Pressure	144.41 ± 4.76	144.14 ± 5.17	0.33	0.74
Diastolic Blood Pressure	94.19 ± 3.98	93.45 ± 3.21	1.25	0.21
Hemoglobin	11.47 ± 0.44	10.31 ± 0.40	-16.74	<0.001

Comparison of maternal and newborn outcomes revealed a significant difference in the odds i.e., the odds of developing preeclampsia (OR= 0.051; 95% CI [0.009,0.281], p<0.001), maternal complications (OR= 0.40; 95% CI [0.169, 0.951], p<0.001), and admission of newborn to NICU (OR= 0.46; 95% CI [0.223,0.887], p<0.001) were lower in PIH women without anemia than in PIH women with anemia, whereas, having a preterm baby (OR= 4.70; 95% CI [0.980, 22.536], P=0.036) and low birth weight baby (OR= 0.21; 95% CI [0.100, 0.434], p<0.001) were higher in PIH with anemia mothers. Indeed, having normal vaginal delivery (OR= 0.382; 95% CI [0.154, 0.950], p = 0.03) was lower in pregnant women with PIH and anemia than in those without anemia (Table 3). The demographic characteristics of the participants showed no significant influence on BMI, BP, and Hb (Table 1).

**Table 3:** Comparison of maternal and newborn outcomes * Chi-square test, †Independent sample t-test, OR: Odds Ratio, CI: Confidence Interval

Outcome Variables		PIH without Anemia (n=73)	PIH with Anaemia (n=77)	OR (95%CI)	Test Statistics	p-value
		f (%)	f (%)			
Developed preeclampsia	No	72 (99%)	60 (78%)	0.049 [0.006- 0.379]	15.22*	<0.001
	Yes	1 (1%)	17 (22%)			
Mode of Delivery	Normal Vaginal Delivery	17 (23%)	8 (10%)	0.382 [0.154- 0.950]	4.489*	0.03 (< 0.05)
	Cesarean Section	56 (77%)	69 (90%)			
Maternal complications	No	64 (88%)	49 (64%)	0.40 [0.169- 0.951]	11.65*	<0.001
	Yes	9 (12%)	28 (36%)			
Gestational week of baby	Preterm	2 (3%)	9 (12%)	4.70 [0.980- 22.536]	4.416*	0.036 (< 0.05)
	Normal	71 (97%)	68 (88%)			
Birth weight of newborn	Normal	59 (81%)	36 (47%)	0.21 [0.100- 0.434]	4.301†	<0.001
	Low Birth Weight	14 (19%)	41 (53%)			
	Mean ±SD	2.72 ± .407	2.44 ± .398			
Admissions to the NICU	No	54 (74%)	43 (56%)	0.46 [0.223-0.887]	5.39*	0.020 (< 0.05)
	Yes	19 (26%)	34 (44%)			
Neonatal Mortality	No	71(97%)	75 (97%)	0.06 [0.145-7.07]	0.003*	0.957
	Yes	2 (3%)	2 (3%)			
Systolic Blood Pressure after delivery		117.93 ± 5.21	120.17 ± 5.08	-	-2.663†	0.009 (< 0.05)
Diastolic Blood Pressure after delivery		74.58 ± 5.41	75.45 ± 5.35	-	-1.001†	0.318
APGAR (at 1 minute)		7.58 ± 1.74	7.47 ± 1.63	-	0.395†	0.693
APGAR (at 5 minutes)		8.32 ± 1.67	8.26 ± 1.58	-	0.209†	0.835

## Discussion

The magnitude of PIH with anemia was 51.3% in our study. We found that the PIH accompanied by anemia increases the risk of developing preeclampsia which is a serious condition causing adverse pregnancy outcomes. This finding aligns with research, conducted in Sudan, which also revealed a relation between the anemia severity and probability of preeclampsia in pregnancy [14]. Another study from Ethiopia revealed that women with anemia had a higher likelihood of experiencing an increased incidence of preeclampsia [16]. A study conducted in Iran revealed significant associations between hemoglobin levels during different stages of pregnancy and specific pregnancy complications. Low levels of hemoglobin till 20 weeks of pregnancy are linked to preeclampsia, while low levels in the second half increase the risk of premature rupture of membranes (PROM). Also, mothers with lower blood dilution during pregnancy are more susceptible to preeclampsia. However, hemoglobin levels in both halves of pregnancy predict PROM and preeclampsia, whereas increased hematocrit levels in the second half or no reduction between the two halves may indicate preeclampsia [17]. A study confirmed that preeclampsia correlates with iron deficiency anemia, leading to elevated pro-inflammatory cytokines and lactoferrin, especially in individuals with moderate to severe anemia [18].

**Figure 1:**
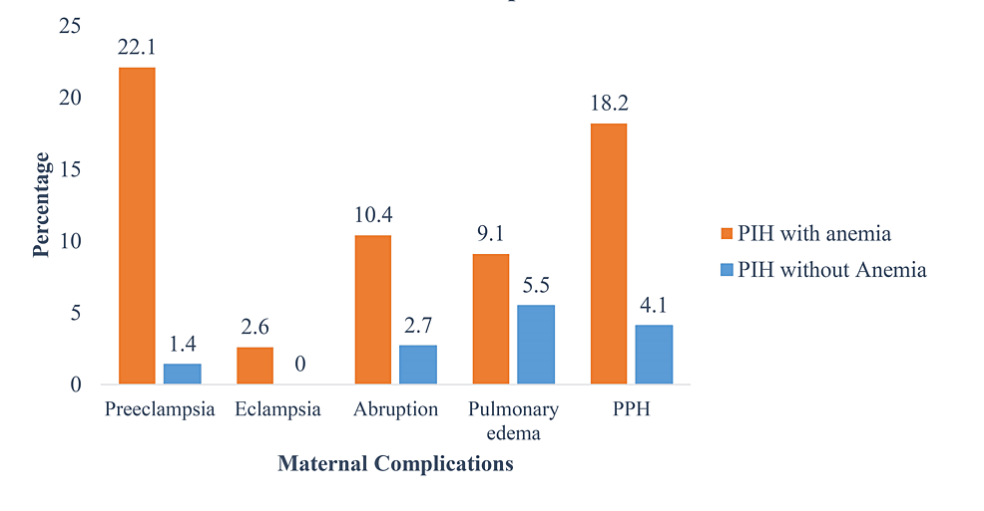
Maternal complications among pregnant women in PIH with anemia and PIH without anemia PIH: Pregnancy-induced hypertension

Our research findings indicate that women experiencing both PIH with anemia have a likelihood of undergoing cesarean sections compared to those with PIH alone. This association was similarly observed in a study conducted by Gupta [19]. The heightened probability of cesarean delivery in these cases might be attributed to the increased risk of complications resulting from the coexistence of both conditions, necessitating surgical intervention during childbirth. However, it should be noted that the available evidence on the combined effect of maternal anemia and PIH on the risk of cesarean delivery remains inconclusive. We found that women with PIH accompanied by anemia experienced more maternal complications during their pregnancy and childbirth such as eclampsia, abruption, pulmonary edema, and postpartum hemorrhage (PPH) (Figure 1).

In this study, we also found that participants with PIH accompanied by anemia were more likely to deliver preterm (12%) and low birth weight (53%) babies and be admitted in the NICU. Similar findings were reported in previous research studies. A study from Madhya Pradesh reported significantly higher maternal complications among pregnant women with both PIH and anemia [19]. A study conducted in China demonstrated that having severe or moderate anemia in pregnancy caused abruptio placenta, severe PPH, even maternal and neonatal death [20]. A comprehensive review of 148 studies found that maternal anemia was a predictor of adverse outcomes, including newborn and maternal mortality [21]. Likewise, a study in Gambia revealed that severe maternal anemia was linked with stillbirths, low birth weight, NICU admissions, and preterm deliveries [22]. Maternal anemia also showed adverse effects such as PIH in pregnant women from Nepal, along with PPH, and had admissions to intensive care unit [23]. Similarly, Pakistani and Indian pregnant women who were severely anemic were found to be encountered with complications like stillbirth, preterm delivery, low birth weight, and postpartum hemorrhage [24]. Many other studies also reported that anemia in pregnancy results in maternal morbidity [14,24-26].

The possible reason could be anemia can lead to reduced oxygen-carrying capacity in the blood, which could exacerbate the impact of PIH on maternal health and increase the likelihood of complications during pregnancy [26]. Scientific evidence from molecular and cellular studies supports the idea of correlation between anemia in pregnancy and the risk of mortality associated with PPH. Lower hemoglobin levels were linked to elevated production of nitric oxide (NO) which has a substantial role in declining uterine muscle activity [27]. The involvement of NO in responding to acute hypoxia by inducing hypoxia-inducible factor. Notably, in IUGR or preeclampsia-complicated pregnancies, there was an elevated expression of endothelial NOS (eNOS), potentially representing inadequate placental implantation [28]. The present study showed that after giving birth, women with PIH and anemia showed an increase in SBP compared to those with PIH alone.

The present study showed that after giving birth, women with PIH and anemia had an increase in SBP compared to those with PIH alone. Though the current study findings suggested the interplay of these two conditions on SBP after delivery, the exact mechanism for increased blood pressure is unidentified. However, our study also found that the sociodemographic characteristics, history of hypertension in the family, and the dietary pattern did not show statistical association with anemia and PIH which could be due the limited sample numbers.

The key strength of this study is that it gains greater implication due to the scarcity of literature with integrated evidence concerning the correlation between PIH and anemia, despite their crucial impact on the health of mothers universally. Furthermore, the lack of a multicenter approach, randomization in sampling, and limited background information from the records limit the generalizability of the study findings. However, this study highlighted the need for further research into the underlying mechanisms to identify the linkages between PIH, anemia, and its outcome. Also, research is needed to evaluate the various interventions in mitigating their combined effects and to improve the pregnancy outcomes. 

## Conclusions

This study provides valuable insights into the pregnancy outcomes of pregnant women with PIH accompanied by anemia and those with PIH alone which call for vigilant monitoring to address anemia in conjunction with PIH. Also, the findings have the potential to influence public health initiatives and policies related to maternal and child health. As we delve into the intricate interplay between anemia and PIH, we acknowledge the importance of comprehensive maternal care and the collaboration between healthcare providers, researchers, and pregnant individuals. By deepening our understanding of the outcomes from these coexisting conditions, we strive to pave the way to improve management strategies for better pregnancy outcomes.
